# The Talbot Effect for two-dimensional massless Dirac fermions

**DOI:** 10.1038/srep26698

**Published:** 2016-05-25

**Authors:** Jamie D. Walls, Daniel Hadad

**Affiliations:** 1 Department of Chemistry, University of Miami, Coral Gables, Florida 33124, USA

## Abstract

A monochromatic beam of wavelength *λ* transmitted through a periodic one-dimensional diffraction grating with lattice constant *d* will be spatially refocused at distances from the grating that are integer multiples of 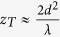. This self-refocusing phenomena, commonly referred to as the Talbot effect, has been experimentally demonstrated in a variety of systems ranging from optical to matter waves. Theoretical predictions suggest that the Talbot effect should exist in the case of relativistic Dirac fermions with *nonzero* mass. However, the Talbot effect for massless Dirac fermions (**mDf**s), such as those found in monolayer graphene or in topological insulator surfaces, has not been previously investigated. In this work, the theory of the Talbot effect for two-dimensional **mDf**s is presented. It is shown that the Talbot effect for **mDf**s exists and that the probability density of the transmitted **mDf**s waves through a periodic one-dimensional array of localized scatterers is also refocused at integer multiples of *z*_*T*_. However, due to the spinor nature of the **mDf**s, there are additional phase-shifts and amplitude modulations in the probability density that are most pronounced for waves at non-normal incidence to the scattering array.

In 1836, H. F. Talbot discovered that the intensity of light transmitted through a periodic grating exhibits a “self-imaging” of the grating at integer multiples of the distance 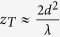 away from the scattering array, where *λ* is the wavelength of light and *d* is the grating’s lattice constant^1^. This self-refocussing of the scattered light intensity is now referred to as the Talbot effect. As first explained by Lord Rayleigh^2^, the Talbot effect is the result of constructive interference of a coherent wave scattered from a periodic array. Within the realm of optical physics, the Talbot effect has been used in a variety of applications in nanolithography^3^, optical metrology and imaging^4^, and light field sensors^5^. The Talbot effect has also been observed in experiments on matter waves^6^, electron beams^7^,^8^, plasmonic devices^9^,^10^, wave guides, and in photonic crystals^11^, along with a recent proposal^12^ to look at a spin Talbot effect in a two-dimensional electron gases (2DEG).

Sir Michael Berry was the first to make a deeper connection between the physics of the Talbot effect and that of quantum revivals observed for confined quantum particles^13^,^14^,^15^, where an initial quantum wave packet undergoes spatiotemporal refocussing as a result of quantum interference. With the discovery of new materials that possess electronic structures that can be described by the relativistic Dirac equation, such as monolayer graphene^16^ and the two-dimensional surface states of topological insulators^17^,^18^,^19^ such as Bi_2_Se_3_, theoretical extensions of the Talbot effect to the case of relativistic quantum revivals were also performed^20^,^21^,^22^ where it was shown that under certain conditions, bound relativistic particles with *nonzero* mass could also exhibit spatiotemporal revivals. From this theoretical work, however, it was not clear whether quantum revivals or, for that matter, the Talbot effect could exist for *massless* Dirac fermions (**mDf**s) since confining such particles is difficult due to Klein tunneling^23^,^24^. While recent numerical calculations^25^ have shown that a Talbot effect can be present in two-dimensional phononic crystals with a dispersion relation that mimics the **mDf** dispersion relation, a full theory of the Talbot effect for **mDf**s is still lacking.

In this paper, we consider the relativistic analogue of Talbot’s original experiment applied to a monochromatic beam of two-dimensional **mDf**s transmitted through a periodic one-dimensional potential. In order to place our theoretical results within a physically realizable context, we consider the particular case of intravalley multiple scattering in monolayer graphene^26^ from a periodic array of localized scatterers as illustrated in Fig. 1. Our previous theoretical work^27^ for the scattering of **mDf** waves from a one-dimensional periodic array of localized scatterers is generalized and used to demonstrate that a Talbot effect exists for **mDf**s. Furthermore, the effects of the **mDf**s’ spinor nature on the predicted Talbot effect is shown to generate an additional amplitude modulation and phase shift in the probability density that is most pronounced for **mDf**s at non-normal incidence to the scattering array.

## Results

**Figure 1:**
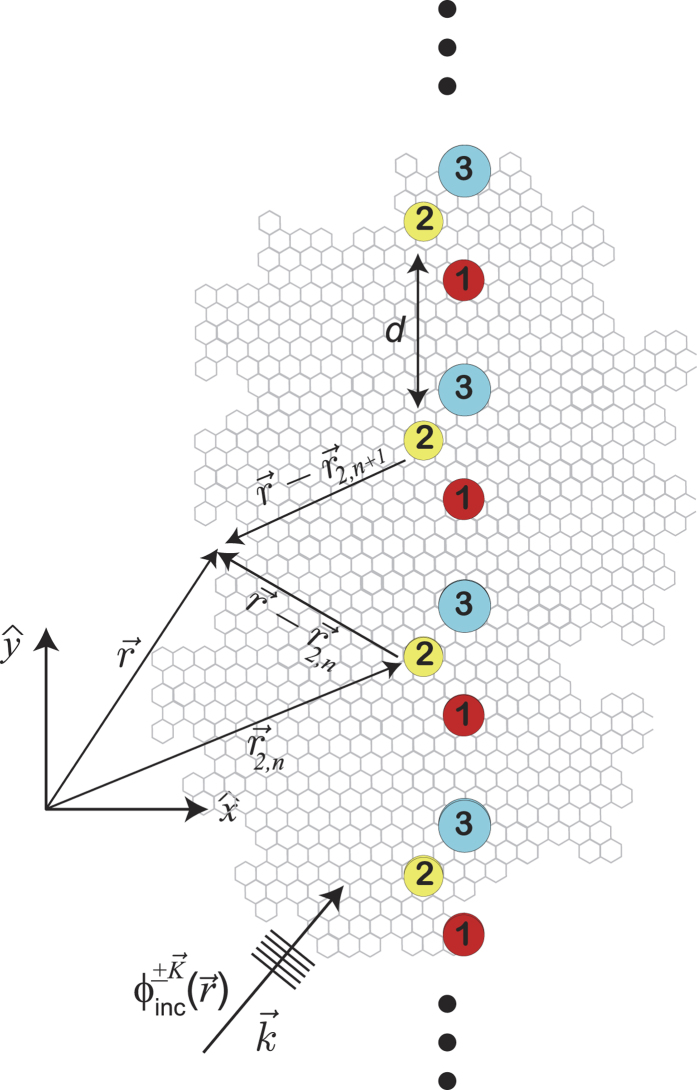
Scattering of an incident **mDf** wave in graphene with energy 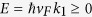, 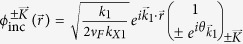, from a one-dimensional array of localized, cylindrically symmetric, nonmagnetic scatterers. In the Figure, the unit cell consists of *N*_*s*_ = 3 localized cylindrically symmetric scatterers. The positions of the scatterers are denoted by 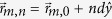 where the subscript 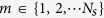 denotes the particular scatterer in the *n*^*th*^ unit cell.

We consider the case of a **mDf** wave in graphene with energy *E* = *ħv*_*F*_*k*_1_ ≥ 0 and wave vector 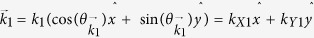, 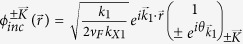, that is incident to a one-dimensional array of localized, cylindrically symmetric, nonmagnetic scatterers as shown in Fig. 1. The subscript, 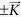, is the valley index and denotes the corresponding Dirac point that the scattering solutions are expanded about. The transmitted wave function to the right of the scattering array (*x* ≫ *d*) is given by:


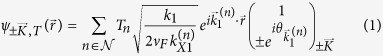


The sum in equation (1) is over all “open” channels denoted by integers 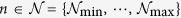 where 
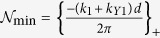
 and 
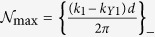
, where {*z*}_+_ corresponds to the smallest integer greater than *z*, and {*z*}_−_ corresponds to the largest integer less than *z*. For 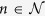, the wave vector associated with the *n*^*th*^ open channel, 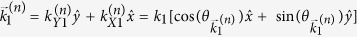 with 
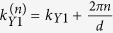
 and 
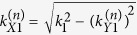
, is purely real. Note that for incident waves with wavelengths 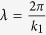 satisfying 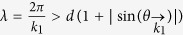, 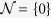 and 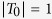. Under these conditions, the incident wave is not scattered by the scattering array and is perfectly transmitted. In Supporting Information, general expressions for the transmission coefficients, *T*_*n*_ in equation (1), are provided.

Writing the transmission coefficient for the *n*^*th*^ open channel as 
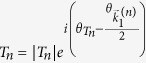
 for *n* ≠ 0 and 
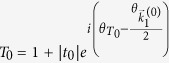
, the dimensionless probability density for *x* ≫ *d*, 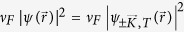, can be written as:


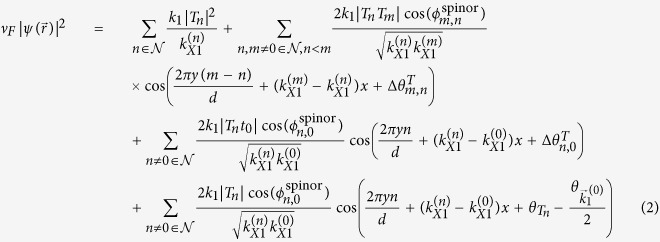


where 
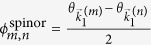
, and 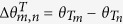. In equation (2), the interference between different open channels contained in 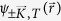 in equation (1) will generate the Talbot effect for **mDf**s, which again requires that 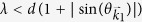 so that *n* ≠ 0 open channels are available to generate an interference pattern. Since the transmission coefficients are independent of the valley index or chirality of the incident wave, the probability density of the transmitted waves is also independent of the chirality of the incident waves.

For comparison, the dimensionless probability density for an achiral electron wave with an effective mass of m* in a 2DEG, 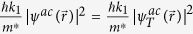, can be written as (see Supporting Information for details):


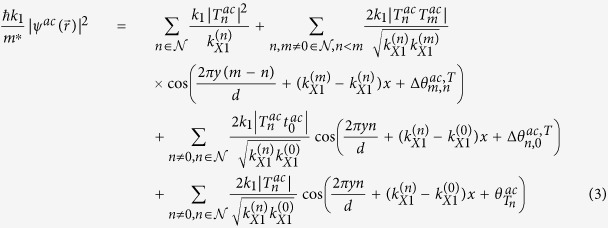


where 
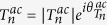
 for *n* ≠ 0 and 
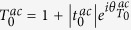
 are the transmission coefficients for the *n*^*th*^ open channel with 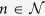, and 
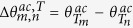
. Comparing equations (2) and (3), the dimensionless probability densities for both **mDf**s and 2DEGs consist of a constant plus a sum over cosine terms that are periodic along both the 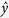
- and 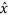-directions with periods 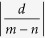 and 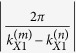 for *m* ≠ *n*, respectively. In particular, for a normally incident wave 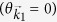 with *λ* < *d*, 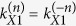, and the periodicity along the 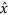-direction in equation (2) and equation (3) can be used to define a set of “Talbot lengths”, 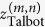 for |*m*| > |*n*| ≥ 0 and 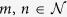, which are given by:


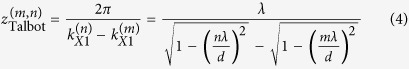


When *λ* ≪ *d*, the paraxial approximation gives 
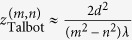
. The traditional Talbot distance defined by Lord Rayleigh^2^ corresponds to 
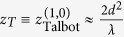
. Thus the Talbot length for **mDf**s and achiral 2DEGs are identical to the traditional Talbot length. Furthermore, similar phase shifts, 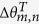 in equation (2) for a **mDf** and 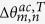 in equation (3) for an achiral 2DEG, are both the result of the scattering potential, which is reminiscent of the phase shifts associated with the Talbot-Beeby effect^28^. However, due to the spinor nature of the **mDf**s, the cosine terms in equation (2) possess an additional amplitude factor of 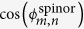, and the last term in equation (2) also has an additional phase shift of 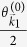 that is due to the interference between the incident wave, 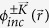, and the 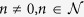 open scattering channels.

Similarly, the reflected wave function, 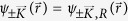 for *x* ≪ −*d*, is given by:


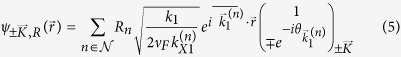


where 
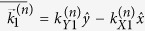
 for 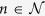. Expressions for the reflection coefficients, 
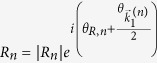
 in equation (5), are given in Supporting Information. The dimensionless probability density to the left of the scattering array, 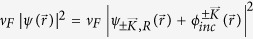 for *x* ≪ −*d*, is given by:


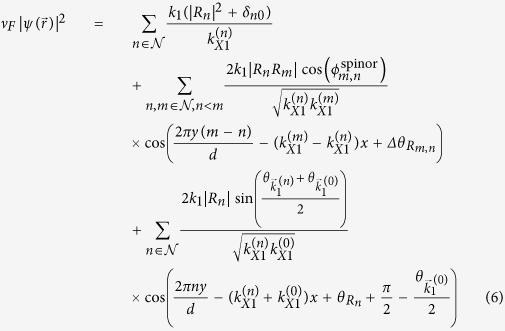


where 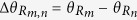. For comparison, a similar calculation of the dimensionless probability density in a 2DEG gives:


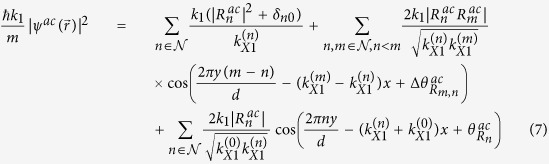


where 
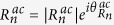
 are the reflection coefficients for the *n*^*th*^ open channel with 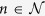 (expressions are given in Supporting Information), and 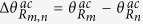. Comparing equations (6) and (7), the dimensionless probability densities for both **mDf**s and 2DEGs consist of a constant plus a sum over cosine terms that are periodic along both the 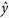
- and 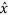-directions with periods either given by 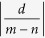 and 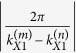 for *m* ≠ *n*, respectively, or by 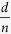 and 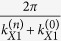 for *n* ≠ 0, respectively. Due to the spinor nature of the **mDf**s, however, there are again additional amplitude factors of 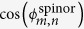 that are identical to those found in equation (2) for the transmitted wave. Furthermore, the interference between the incident wave and the *n* ≠ 0 reflected waves results in an amplitude factor of 
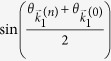
 along with a phase shift of 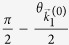 relative to that found in an achiral 2DEG. As a result, a greater difference in the probability densities between **mDf**s and achiral 2DEGs will in general be observed for the reflected waves relative to that found for the transmitted waves.

**Figure 2:**
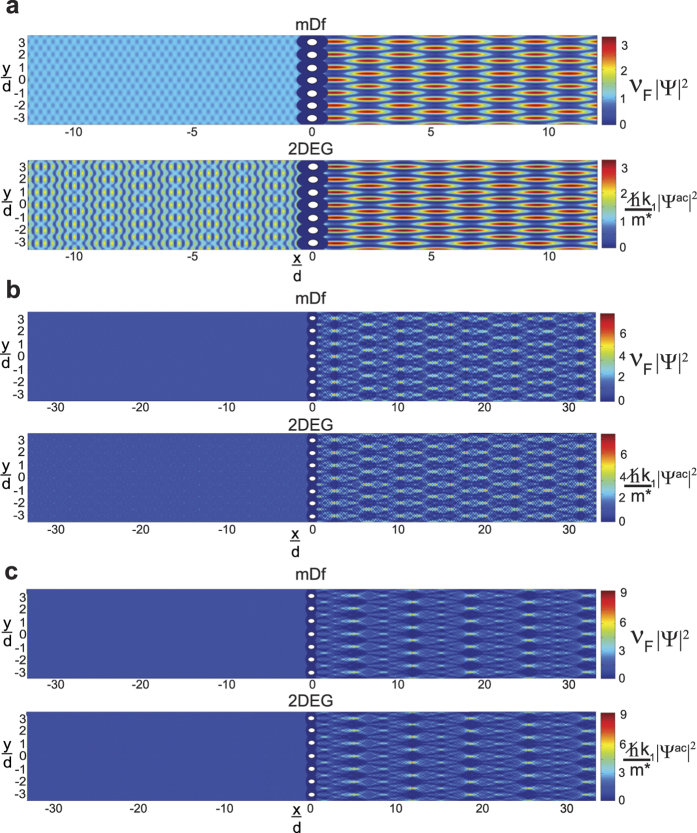
Plots of the dimensionless probability densities in a **mDf**, 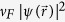, and in an achiral 2DEG, 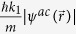, for an electron wave normally incident 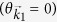 to an infinite one-dimensional array with lattice constant *d* = 30 nm consisting of a single scatterer per unit cell with *r*_*s*_ = 4 nm and *V* = −200 meV at the following wave vector magnitudes and *l*_*max*_: (**a**) 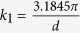 and *l*_*max*_ = 4, (**b**) 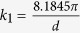 and *l*_*max*_ = 6, and (**c**) 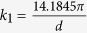 and *l*_*max*_ = 9.

In Fig. 2, numerical calculations of the dimensionless probability densities for a **mDf** and an achiral 2DEG wave normally incident 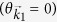 to a one-dimensional array of scatterers with lattice constant *d* = 30 nm and with a unit cell consisting of a single scatter of potential *V* = −0.2 eV and radius *r*_*s*_ = 4 nm are shown. For comparison, *k*_1_ was chosen to be the same in both the **mDf** and the achiral 2DEG in all cases; inside the scattering regions, the magnitude of the wave vector was chosen to be 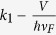 in both the **mDf** and the achiral 2DEG. In the plots of 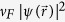 and 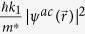 in Fig. 2, the following values of *k*_1_*d, l*_*max*_ + 1 partial waves scattering from a single scatterer, and the open scattering channels 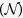 were used in the calculations along with the corresponding total transmission probabilities: (Fig. 2a) *k*_1_*d* = 3.1845*π, l*_*max*_ + 1 = 5, 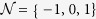, 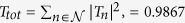, and 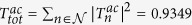, (Fig. 2b) *k*_1_*d* = 8.1845*π, l*_*max*_ + 1 = 7, 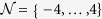, *T*_*tot*_ = 0.9862, and 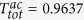, and (Fig. 2c) *k*_1_*d* = 14.1845*π, l*_*max*_ + 1 = 10, 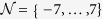, *T*_*tot*_ = 0.9936, and 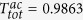. As seen in Fig. 2, similar periodic patterns in the probability density appear to the right of the scattering array in both the **mDf** and achiral 2DEG calculations whereas the 
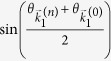
 amplitude factors and phase shifts of 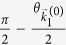 found in the reflected **mDf** probability density in equation (2) lead to large differences in the probability to the left of the scattering array relative to that found in an achiral 2DEG [equation (6) vs. equation (7)]. The difference in probability densities between the **mDF** and an achiral 2DEG was most pronounced for the longest wavelength case 
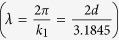
 shown in Fig. 2a due to ***(i)*** the larger difference in reflection probabilities between the **mDf** and achiral 2DEG cases (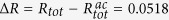 in Fig. 2(a) versus Δ*R* = 0.0225 and Δ*R* = 0.0073 in Fig. 2(b,c), respectively) and ***(ii)*** the fact that the reflected probability is spread out over fewer backscattering channels in the longer wavelength case [*n* ∈ {±1}] relative to the shorter wavelength cases [*n* ∈ {±1, ±2, ±3, ±4} in Fig. 2b and *n* ∈ {±1, ±2, … ±6, ±7} in Fig. 2c].

It is known from previous theoretical^26^,^29^,^30^ and experimental^31^,^32^,^33^,^34^ work that a particle’s spinor nature can significantly affect the observed interference patterns of waves undergoing multiple scattering. However, the observed differences in the probability densities of an **mDf** and achiral 2DEG in Fig. 2 are due not only to the spinor nature of the **mDf**s but also due to differences in transmission and reflection coefficients, *T*_*n*_ and *R*_*n*_ for the **mDf** versus 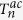 and 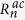 for the achiral 2DEG. Therefore, to isolate the effects of the spinor nature of the **mDf**s on the probability density, we can replace 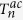 and 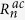 by *T*_*n*_ and *R*_*n*_ in the right hand sides of equation (3) and equation (7) to calculate the probability density for a “spinless” **mDf**, 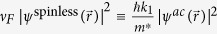. In this case, the relative difference in probability density due solely to the spinor nature of the **mDf**s, *χ*, can be calculated using:


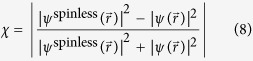


**Figure 3:**
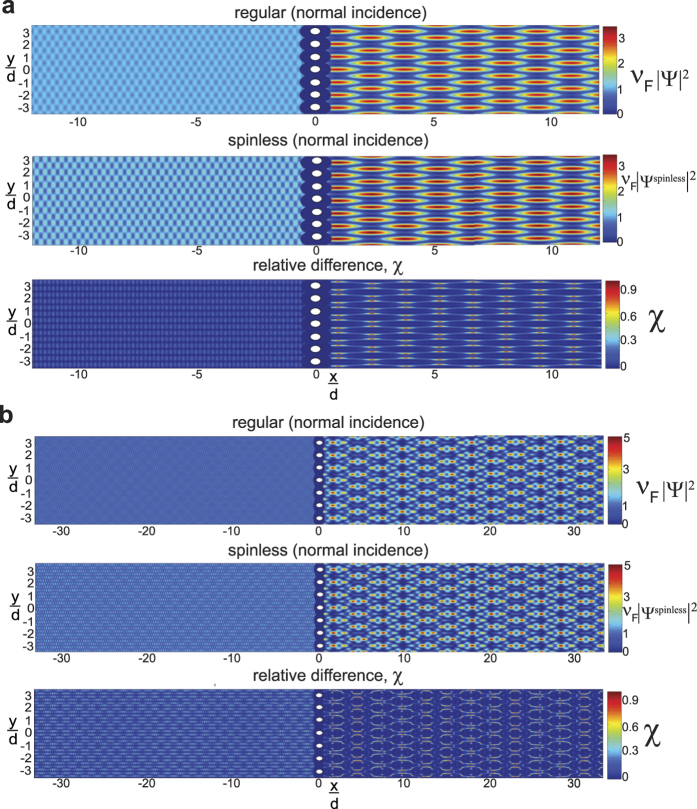
Plots of the dimensionless probability densities in a regular mDf, 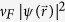 and in a spinless mDf, 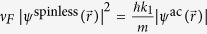 (equations 3 and 7 with 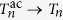 and 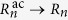) along with their relative probability differences *χ* [equation (8)]. Calculations were performed for waves at normal incidence 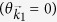 to an infinite one-dimensional array with lattice constant *d* = 30 nm consisting of a single scatterer per unit cell with *r*_*s*_ = 4 nm and *V* = −200 meV at the following wave vector magnitudes, *T*_*tot*_, and *l*_*max*_: (**a**) 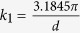, *T*_*tot*_ = 0.9867, and *l*_*max*_ = 4 and (**b**) 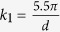, *T*_*tot*_ = 0.9650, and *l*_*max*_ = 6.

**Figure 4:**
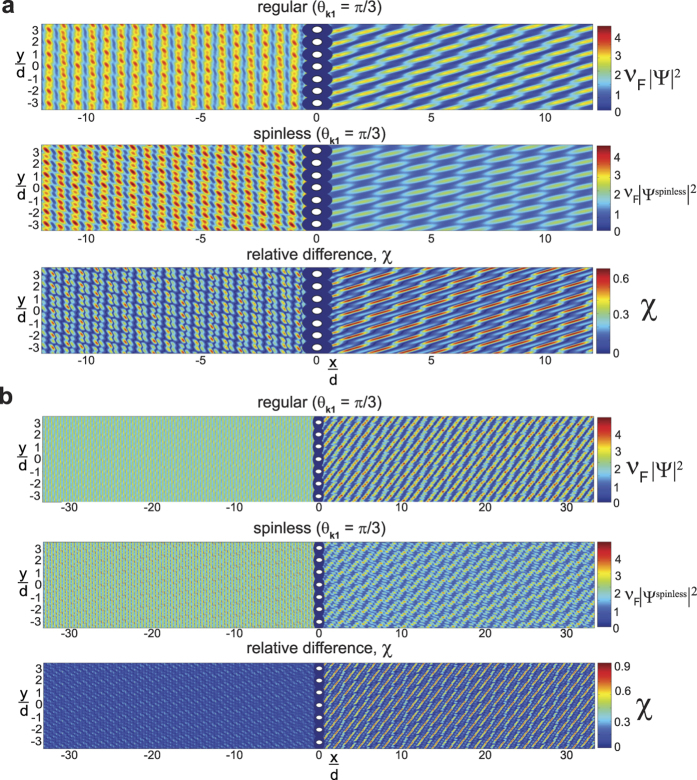
Plots of the dimensionless probability densities in a regular mDf, 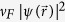 and in a spinless mDf, 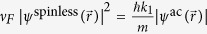 (equations 3 and 7 with 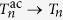 and 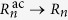) along with their relative probability differences *χ* (equation (8)). Calculations were performed for waves at non-normal incidence 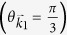 to an infinite one-dimensional array with lattice constant *d* = 30 nm consisting of a single scatterer per unit cell with *r*_*s*_ = 4 nm and *V* = −200 meV at the following wave vector magnitudes, *T*_*tot*_, and *l*_*max*_: (**a**) 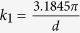, *T*_*tot*_ = 0.8627, and *l*_*max*_ = 4 and (**b**) 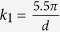, *T*_*tot*_ = 0.9485, and *l*_*max*_ = 6.

Plots of the dimensionless probability densities and relative probability density differences, *χ* [equation (8)], for both regular and “spinless” **mDf**s waves scattering from the same scattering potentials used in Fig. 2 are given in Fig. 3 (at normal incidence, 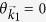) and Fig. 4 (at non-normal incidence, 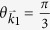). Two different wave vector amplitudes were used in the calculations: *k*_1_*d* = 3.845*π* [Figs 3a and 4a] and *k*_1_*d* = 5.5*π* [Figs 3b and 4b]. At normal incidence (Fig. 3), the relative probability density difference to the right of the scattering array, which is mainly due to the cos(*ϕ*^spinor^) amplitude factors in equation (2), is only significant over a small area. However, at non-normal incidence (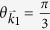 in Fig. 4), the probability densities are significantly different between the normal and “spinless” **mDf**s over a larger area, which is consistent with our theoretical predictions. In this case, the difference in probability density is due not only to the cos(*ϕ*^spinor^) amplitude factors but also the phase shifts generated from the interference between the incident wave and the *n* ≠ 0 “open” transmitted/reflected waves in equations (2) and (6).

**Figure 5:**
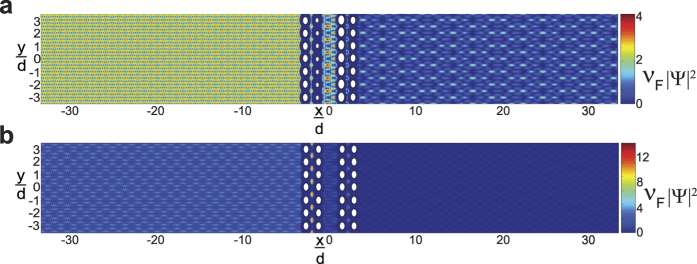
Plots of the dimensionless probability densities in a mDf, 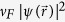 for a wave with *k*_1_*d* = 5.5*π* normally incident 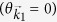 to an infinite one-dimensional array with lattice constant *d* = 30 nm and a unit cell consisting of *N*_*s*_ = 4 scatterers of potential *V* = −330 meV in a collinear arrangement with 
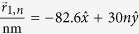
, 
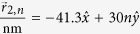
, 
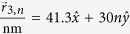
, and 
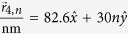
. The scatter sizes were either chosen to be either (**b**) equal with *r*_*s*1_ = *r*_*s*2_ = *r*_*s*3_ = *r*_*s*4_ = 4 nm, which resulted in *T*_*tot*_ = 0.2320 or (**a**) unequal with *r*_*s*1_ = *r*_*s*4_ = 4 nm, *r*_*s*2_ = 2 nm, and *r*_*s*3_ = 6 nm, which resulted in *T*_*tot*_ = 0.3739. In both calculations, *l*_*max*_ = 6 was chosen.

**Figure 6:**
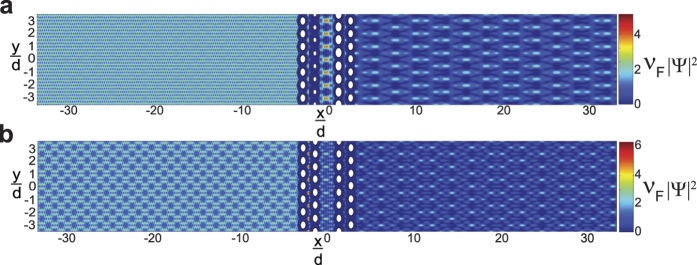
Plots of the dimensionless probability densities in a mDf, 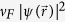 for a wave with *k*_1_*d* = 5.5*π* normally incident 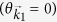 to an infinite one-dimensional array with lattice constant *d* = 30 nm with a unit cell consisting of *N*_*s*_ = 4 scatterers of potential *V* = −330 meV in a nonlinear arrangement with 
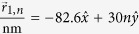
, 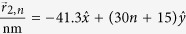, 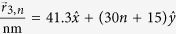, and 
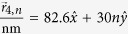
. The scatter sizes were chosen either to be (**b**) equal with *r*_*s*1_ = *r*_*s*2_ = *r*_*s*3_ = *r*_*s*4_ = 4 nm, which resulted in *T*_*tot*_ = 0.4611, or (**a**) unequal with *r*_*s*1_ = *r*_*s*4_ = 4 nm, *r*_*s*2_ = 2 nm, and *r*_*s*3_ = 6 nm, which resulted in *T*_*tot*_ = 0.5514. In both calculations, *l*_*max*_ = 6 was chosen.

While the results in Figs 2, 3, 4 considered a scattering array with a unit cell consisting of a single scatterer, the theory developed in this work can also be applied to arbitrary scatterer configurations within a unit cell. In Figs 5 and 6, 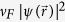 was calculated for a wave with *k*_1_*d* = 5.5*π* that was normally incident (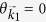) to a scattering array with lattice constant *d* = 30 nm and with a unit cell consisting of four scatterers at potential *V* = −0.33 eV that were either in a collinear arrangement with 
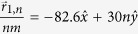
, 
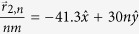
, 
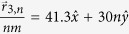
, and 
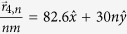
 as shown in Fig. 5 or in a nonlinear arrangement with 
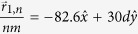
, 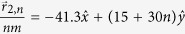, 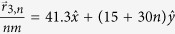, and 
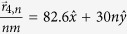
 as shown in Fig. 6. For both scatterer arrangements, the sizes of the scatterers were taken either to be equal [*r*_*s*1_ = *r*_*s*2_ = *r*_*s*3_ = *r*_*s*4_ = 4 nm in Figs 5b and 6b] or unequal [*r*_*s*1_ = *r*_*s*4_ = 4 nm, *r*_*s*2_ = 2 nm, and *r*_*s*3_ = 6 nm in Figs 5a and 6a]. The nonlinear arrangement of scatterers led to larger total transmission probabilities relative to the linear arrangement [*T*_*tot*_ = 0.5514 in Fig. 6a vs. *T*_*tot*_ = 0.3739 in Fig. 5a and *T*_*tot*_ = 0.4611 in Fig. 6b vs. *T*_*tot*_ = 0.232 in Fig. 5b]. For both types of scatterer arrangements, the total transmission probabilities were also larger when the scatterers were of unequal sizes. Finally, although the Talbot lengths, 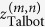 in equation (4), depend solely upon *λ* and *d*, the fine structure in 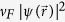 depends sensitively upon the details of the scatterer sizes, potentials, and arrangements within a unit cell, which ultimately determines the various transmission and reflection coefficients, *T*_*n*_ and *R*_*n*_ in equation (2) and equation (6), respectively.

**Figure 7:**
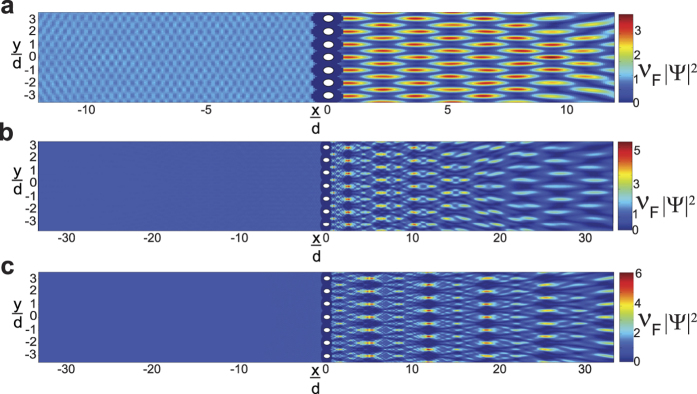
Plots of 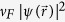 for a normally incident mDf wave 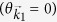 to a *finite* one-dimensional array of 21 identical scatterers with *r*_*s*_ = 4 nm, *V* = −200 meV, and with the position of the *n*^*th*^ scatterer given by 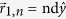 with *d* = 30 nm and *n* ∈ [−10, 10]. The same wave vectors and *l*_*max*_ values used in Fig. 2 were also used for the finite scattering array: (**a**) 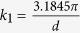 and *l*_*max*_ = 4, (**b**) 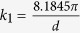 and *l*_*max*_ = 6, and (**c**) 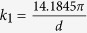 and *l*_*max*_ = 9.

Finally, we consider an incident wave scattering from a *finite* scattering array. In this case, previous theoretical work on multiple scattering from a finite number of scatterers^26^,^27^ was applied to calculate 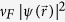. In Fig. 7, 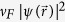 for a wave normally incident to a finite scattering array is shown, where the scattering array consists of *N* = 21 equally spaced cylindrically symmetric scatterers with *r*_*s*_ = 4 nm and *V* = −200 meV that were placed along the 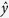-axis between 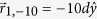 to 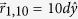 with *d* = 30 nm. The incident wave vectors were chosen to be identical to those used in Fig. 2 to enable a better comparison of 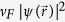 between the finite and infinite scattering arrays. While the overall periodic structures observed in 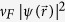 were similar in both the finite [Fig. 7] and infinite [Fig. 2] cases, some of the finer structures/interference patterns observed in the infinite scattering array were absent for the finite scattering array. The periodic structures in the finite case also became blurrier with increasing distance from the scattering array, particularly at distances *x* ≫ 10*d* from the center of the scattering array. This was a consequence of the finite size of the scattering array whereby the interference patterns in 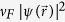 decay approximately as 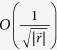. However, at distances within 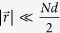 from the center of the scattering array, a clear periodic pattern was still observed in the case of a finite scattering array.

## Discussion

In this work, the theory of the two-dimensional Talbot effect for massless Dirac fermions (**mDfs**) was presented. It was shown that the Talbot effect for **mDf**s exists with Talbot lengths, 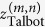 in equation (4), that were identical to those found for an achiral two-dimensional electron gas (2DEG). The interference patterns seen in the Talbot effect are a result of coherent electron transmission of **mDf**s through the scattering array, whereby multiple scattering pathways constructively interfere at distances away from the scattering array determined by the periodicity of the scattering array. However, due to the spinor (or pseudospinor in the case of graphene) nature of **mDf**s, the periodic structures found in the probability density were both amplitude modulated and phase shifted relative to those found in an achiral 2DEG. Such differences were most pronounced for **mDf** waves at non-normal incidence to the scattering array. Numerical calculations on *finite* scattering arrays demonstrated that periodic structures in the probability density still exist but that these structures decay with increasing distance from the scattering array. While the probability density is independent of which valley point the scattering states are expanded about [equations (2) and (6)], the use of magnetic scatterers could potentially be used to distinguish the chirality (or in this case, valley index 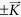) of the incident waves in monolayer graphene. The **mDf** Talbot effect predicted in this work should be observable in systems like monolayer graphene and on the surfaces of topological insulators, where phase coherence lengths greater than 5 *μ*m and 1 *μ*m have been experimentally observed in graphene^35^ and topological insulators^36^, respectively. Overall, this work provides yet another example of the fruitful analogy between traditional optics and coherent “electron” optics in graphene and similar systems^37^,^38^,^39^,^40^.

While there exist proposals^9^,^10^ to employ the Talbot effect for nonrelativistic electrons in plasmonic devices, the theory presented in this work could be used as a starting point for designing and understanding the Talbot effect in graphene and topological insulator^41^,^42^ plasmonic devices. It should also be noted that only coherent dynamics was considered in this work. Spatial and spin/pseudospin decoherence, however, will attenuate and destroy the Talbot effect with increasing distance from the scattering array. As a result, comparing the observed spatial decay of the interference patterns in the Talbot carpet with the interference patterns calculated using the theory presented in this work could provide valuable information about both spatial decoherence^43^ and spin/pseudospin decoherence in two-dimensional **mDf**s.

## Methods

The basic results for intravalley scattering of a plane wave incident to a one-dimensional array of localized scatterers in graphene (as illustrated in Fig. 1) and in a 2DEG are derived in Supporting Information^26^,^27^,^44^,^45^,^46^. The overall theoretical formalism used in this paper represents a generalization of the case of a single scatterer per unit cell^27^ to the case of multiple scatterers within a unit cell. From Fig. 1, the incident waves with energy 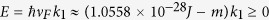, which are labeled by the corresponding valley index or Dirac point that the plane wave states are expanded about in graphene, 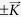, are given by 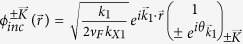, where 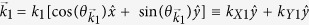 with 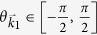 for waves incident to the scattering array from the left. The unit cell of the scattering array consists of *N*_*s*_ localized cylindrically symmetric scatterers with a lattice constant *d*. The overall scattering potential can be written as 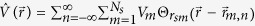 where 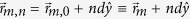 denotes the position of the *m*^*th*^ scatterer in the *n*^*th*^ unit cell, *V*_*m*_ and *r*_*sm*_ are the potential and radius of the *m*^*th*^ scatterer, respectively, and 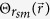 is Heaviside step function given by:


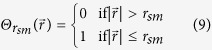


In this work, the potentials of the individual scatterers were taken to be identical in order to avoid the confounding effects of electric fields between the scatterers, i.e., *V*_*m*_ = *V* for all *m* ∈ {1, 2, …, *N*_*s*_}. The *l*^*th*^ partial wave scattering amplitude from the *m*^*th*^ cylindrically symmetric scatterer, *s*_*m,l*_ with 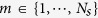, is given by^26^,^46^:


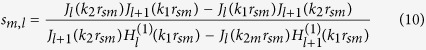


where 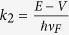 is the magnitude of the wave vector inside scatterer regions, and *J*_*l*_(*z*) is a bessel function of the first kind of order *l*, respectively. For the *n*^*th*^ scatterer with 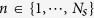 *l*_*max,n*_ + 1 partial waves were chosen to account for greater than 99.9999% of the total scattering amplitude, i.e., 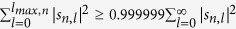. For *N*_*s*_ scatterers within a unit cell, *l*_*max*_ is just the maximum partial wave needed to take into account at least 99.9999% of the total scattering amplitude from *all* scatterers, i.e., 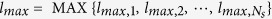. Derivations of the scattering solutions for both a **mDf** and a 2DEG are given in Supporting Information. Finally, all calculations shown in Figs 2, 3, 4, 5, 6, 7 were carried out using in-house MATLAB (Mathworks) programs.

## Additional Information

**How to cite this article**: Walls, J. D. and Hadad, D. The Talbot Effect for two-dimensional massless Dirac fermions. *Sci. Rep.* **6**, 26698; doi: 10.1038/srep26698 (2016).

## Supplementary Material

Supplementary Information
