# Supplementary material for: The Talbot Effect for two-dimensional massless Dirac fermions
Source: Sci Rep. 2016 May 25;6:26698. doi: 10.1038/srep26698 (PMC4879638; doi:10.1038/srep26698)
Supplement: Supplementary Information [file srep26698-s1.pdf]

Supporting Information for "The Talbot Effect for two-dimensional massless Dirac fermions"

Jamie D. Walls[1,*] and Daniel Hadad[1]

[1]*Department of Chemistry, University of Miami, Coral Gables, Florida 33124,USA*

[*] Corresponding author: jwalls@miami.edu

In the following, the basic theory for intravalley multiple scattering of massless Dirac fermions (**mDf**s) is presented and applied to the problem of a plane wave scattering from an infinite, one-dimensional array of localized scatterers. First, the basic formalism for calculating the scattering solutions for a plane wave scattered from a finite number of localized scatterers in graphene is presented. Next, the scattering wave functions, $\psi_{\pm\vec{K}}(\vec{r})$, for the single and two scatterer cases are explicitly presented. The theory is then extended to the case of scattering from an infinite, one-dimensional array of localized scatterers in graphene where explicit expressions for the reflection and transmission coefficients are provided. Finally, the analogous theory for scattering from an infinite, one-dimensional array of localized scatterers in a two-dimensional electron gas (2DEG) is derived for comparison to the graphene results.

I. CALCULATIONS OF THE SCATTERING STATES FROM AN INFINITE ONE-DIMENSIONAL ARRAY OF LOCALIZED SCATTERERS IN GRAPHENE, $\psi_{\pm\vec{K}}(\vec{r})$

In the following, we extend the basic formalism for intravalley multiple scattering in graphene[1] applied to scattering from a periodic, one-dimensional scattering array of localized scatterers with a unit cell consisting of a single scatterer[2] to a unit cell consisting of N_s localized cylindrically symmetric scatterers as shown in Figure 1, i.e., $\widehat{V}(\vec{r}) = \sum_{n=-\infty}^{\infty}\sum_{m=1}^{N_s} V_m \Theta_{r_{sm}}(\vec{r}-\vec{r}_{m,n})$ where $\vec{r}_{m,n} = \vec{r}_{m,0} + nd\widehat{y} \equiv \vec{r}_m + nd\widehat{y}$ denotes the position of the mth scatterer in the nth unit cell, V_m and r_{sm} are the mth scatterers potential and radius, respectively, and $\Theta_{r_{sm}}(\vec{r})$ is the Heaviside step function given by:

$$\Theta_{r_{sm}}(\vec{r}) = \begin{cases} 0 & \text{if} |\vec{r}| > r_{sm} \\ \\ 1 & \text{if} |\vec{r}| \leq r_{sm} \end{cases} \tag{1}$$

In this work, the potentials of the individual scatterers are taken to be identical in order to avoid the confounding effects of electric fields between the scatterers, i.e., $V_m = V$ for all $m \in \{1, 2, ..., N_s\}$.

For intravalley scattering, the scattering solutions are expanded about either the $+\vec{K}$ or $-\vec{K}$ Dirac points, where $\vec{K} = \frac{4\pi\sqrt{3}}{9b}\widehat{x}$ and $b = 1.42\mathring{A}$ is the carbon-carbon bond length in graphene. Let $\phi_{inc}^{\pm\vec{K}}(\vec{r})$ be an incident Dirac plane wave spinor of energy $E = \hbar v_F k_1 = \frac{h v_F}{\lambda}$ normalized to unit flux along the $\widehat{x}$-direction, $\phi_{inc}^{\vec{K}}(\vec{r}) = \sqrt{\frac{k_1}{2v_F k_{X1}}} e^{\vec{k}_1\cdot\vec{r}} \begin{pmatrix} 1 \\ e^{i\theta_{\vec{k}_1}} \end{pmatrix}_{\vec{K}}$ or $\phi_{inc}^{-\vec{K}}(\vec{r}) = \sqrt{\frac{k_1}{2v_F k_{X1}}} e^{\vec{k}_1\cdot\vec{r}} \begin{pmatrix} 1 \\ -e^{i\theta_{\vec{k}_1}} \end{pmatrix}_{-\vec{K}}$

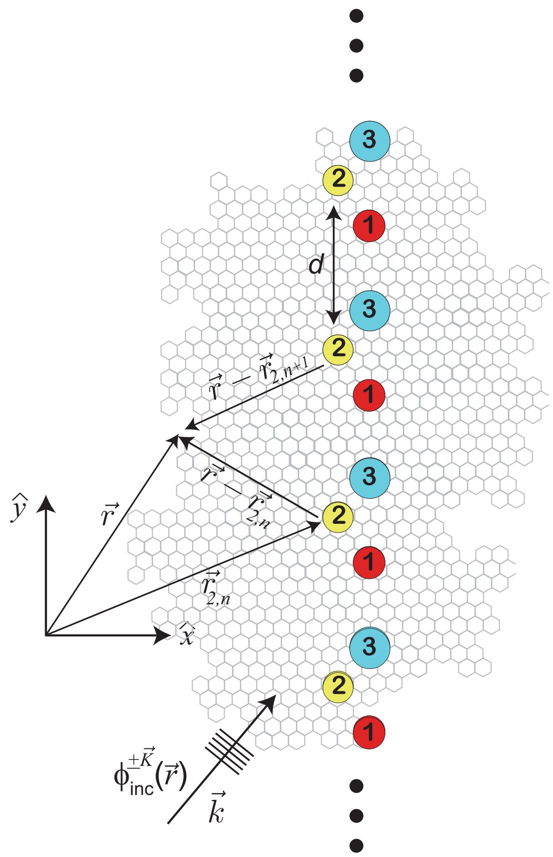

FIG. 1. Scattering of an incident **mDf** wave in graphene with energy $E = \hbar v_F k_1 \geq 0$, $\phi^{\pm\vec{K}}_{inc}(\vec{r}) = \sqrt{\frac{k_1}{2v_F k_{X1}}} e^{i\vec{k}_1\cdot\vec{r}} \begin{pmatrix} 1 \\ \pm e^{i\theta_{\vec{k}_1}} \end{pmatrix}_{\pm\vec{K}}$, from a one-dimensional array of localized, cylindrically symmetric, nonmagnetic scatterers. In the Figure, the unit cell consists of $N_s = 3$ localized cylindrically symmetric scatterers. The positions of the scatterers are denoted by $\vec{r}_{m,n} = \vec{r}_{m,0} + nd\hat{y}$ where the subscript $m \in \{1, 2, \cdots N_s\}$ denotes the particular scatterer in the nth unit cell.

with a wave vector given by $\vec{k}_1 = k_1 \cos(\theta_{\vec{k}_1})\hat{x} + k_1 \sin(\theta_{\vec{k}_1})\hat{y} = k_{X1}\hat{x} + k_{Y1}\hat{y}$ for $\theta_{\vec{k}_1} \in \left(-\frac{\pi}{2}, \frac{\pi}{2}\right)$, and $\hbar v_F = 1.0558 \times 10^{-28}$ J-m.

The total wave function, $\psi_{\pm\vec{K}}(\vec{r})$, can be written as:

$$\psi_{\pm\vec{K}}(\vec{r}) = \phi^{\pm\vec{K}}_{inc}(\vec{r}) + \sum_{m=1}^{N_s} \sum_{l=0}^{l_{max}} \sum_{n=-\infty}^{\infty} \frac{4i\hbar v_F}{k_1} s_{m,l} \widehat{G}_{l,\pm\vec{K}}(\vec{r}-\vec{r}_{m,n}; E) e^{indk_{Y1}} \widehat{T}_{l,\pm\vec{K}} \psi_{\pm\vec{K}}(\vec{r}_{m,0}) \qquad (2)$$

where $s_{m,l}$ is the scattering amplitude of the lth partial wave from scatterer $m \in [1, N_s]$, $l_{max}+1$ are the number of partial waves that are included in calculation of $\psi_{\pm\vec{K}}(\vec{r})$ in Eq. (2) with $l_{max} \geq 0$, and

$$\begin{aligned}\widehat{G}_{l,\pm\vec{K}}(\vec{\Delta r}; E) &= -\frac{i^{l+1}k_1}{4\hbar v_F} \begin{pmatrix} H^{(1)}_l(k_1\Delta r)e^{il\theta_{\vec{\Delta r}}} & \pm iH^{(1)}_{l+1}(k_1\Delta r)e^{-i(l+1)\theta_{\vec{\Delta r}}} \\ \pm iH^{(1)}_{l+1}(k_1\Delta r)e^{i(l+1)\theta_{\vec{\Delta r}}} & H^{(1)}_l(k_1\Delta r)e^{-il\theta_{\vec{\Delta r}}} \end{pmatrix}_{\pm\vec{K}} \\ &= -\frac{ik_1}{4\hbar v_F} \begin{pmatrix} \hat{L}^l_+[H^{(1)}_0(k_1\Delta r)] & \pm\hat{L}^{l+1}_-[H^{(1)}_0(k_1\Delta r)] \\ \pm\hat{L}^{l+1}_+[H^{(1)}_0(k_1\Delta r)] & \hat{L}^l_-[H^{(1)}_0(k_1\Delta r)] \end{pmatrix}_{\pm\vec{K}} \end{aligned} \qquad (3)$$

where $H^{(1)}_l(z)$ is a hankel function of order l, $\Delta r = \left|\vec{\Delta r}\right|$, $e^{\pm i\theta_{\vec{\Delta r}}} = \frac{\vec{\Delta r}\cdot(\hat{x}\pm i\hat{y})}{\Delta r}$, and $\widehat{T}_{l,\pm\vec{K}}$ is the l-partial

wave $t-$matrix operator given by:

$$\widehat{T}_{l,\pm\vec{K}} = \begin{pmatrix} \hat{L}_-^l & 0 \\ 0 & \hat{L}_+^l \end{pmatrix}_{\pm\vec{K}} \tag{4}$$

where $\hat{L}_\pm = \frac{1}{ik_1} e^{\pm i\theta} \left(\frac{\partial}{\partial_r} \pm \frac{i}{r} \frac{\partial}{\partial_\theta} \right) = \frac{1}{ik_1} \left(\frac{\partial}{\partial_x} \pm i \frac{\partial}{\partial_y} \right)$, and $\widehat{T}_{0,\pm\vec{K}} = \widehat{1}$ is the 2×2 identity matrix. In writing Eq. (2), the translational symmetry of the scattering potential, $\widehat{V}(\vec{r}+nd\widehat{y}) = \widehat{V}(\vec{r})$, implies that $\psi_{\pm\vec{K}}(\vec{r}+nd\widehat{y}) = \psi_{\pm\vec{K}}(\vec{r})e^{ik_{Y1}nd}$, which means that only $\psi_{\pm\vec{K}}(\vec{r})$ between $-\frac{d}{2} \le y \le \frac{d}{2}$ needs to be calculated. In Eq. (2), the lth partial wave scattering amplitudes for the mth cylindrically symmetric scatterer, $s_{m,l}$ with $m \in [1, N_s]$, are given[1, 3] by:

$$s_{m,l} = \frac{J_l(k_{2,m}r_{sm})J_{l+1}(k_1 r_{sm}) - J_l(k_1 r_{sm})J_{l+1}(k_{2,m}r_{sm})}{J_{l+1}(k_{2,m}r_{sm})H_l^{(1)}(k_1 r_{sm}) - J_l(k_{2,m}r_{sm})H_{l+1}^{(1)}(k_1 r_{sm})} \tag{5}$$

where $k_{2,m} = \frac{E-V_m}{\hbar v_F}$ is the magnitude of the wave vector inside scatterer m, and $J_l(z)$ is a bessel function of the first kind of order l, respectively.

Knowledge of $\widehat{T}_{l,\pm\vec{K}}\psi_{\pm\vec{K}}(\vec{r}_{m,0})$ for $m \in [1, ..., N_S]$ and $l \in [0, ..., l_{max}]$ completely determines $\psi_{\pm\vec{K}}(\vec{r})$ in Eq. (2); these can be determined self-consistently from the following:

$$\begin{aligned} \widehat{T}_{l',\pm\vec{K}}\psi_{\pm\vec{K}}(\vec{r}_{m,0}) &= \widehat{T}_{l',\pm\vec{K}}\phi_{inc}^{\pm\vec{K}}(\vec{r}_{m,0}) + \sum_{l=0}^{l_{max}}\sum_{n\neq 0} s_{m,l}\left[\widehat{T}_{l',\pm\vec{K}}\widehat{G}_{l,\pm\vec{K}}(-nd\widehat{y};E)\right]e^{ink_{Y1}d}\widehat{T}_{l,\pm\vec{K}}\psi_{\pm\vec{K}}(\vec{r}_{m,0}) \\ &+ \sum_{p\neq m}\sum_{l=0}^{l_{max}}\sum_{n=-\infty}^{\infty} s_{p,l}\left[\widehat{T}_{l',\pm\vec{K}}\widehat{G}_{l,\pm\vec{K}}(\Delta\vec{r}_{mp} - nd\widehat{y};E)\right]e^{ink_{Y1}d}\widehat{T}_{l,\pm\vec{K}}\psi_{\pm\vec{K}}(\vec{r}_{p,0}) \end{aligned} \tag{6}$$

Eq. (6) gives a total of $2N_s(l_{max}+1)$ sets of equations, which can be written compactly as:

$$\left(\widehat{\widehat{\mathbf{1}}} - \widehat{\widehat{\mathbf{TG}}}_{\pm\vec{K}}(k_1 d, k_{Y1}d)\right)\widehat{\widehat{\mathbf{T}}}\widehat{\psi}_{\pm\vec{K}}(cell_0) = \widehat{\widehat{\mathbf{T}}}\widehat{\phi}_{inc}^{\pm\vec{K}}(cell_0) \tag{7}$$

where $\widehat{\widehat{\mathbf{1}}}$ is a $2N_s(l_{max}+1)\times 2N_s(l_{max}+1)$ identity matrix, $\widehat{\widehat{\mathbf{T}}}\widehat{\psi}_{\pm\vec{K}}(cell_0)$ and $\widehat{\widehat{\mathbf{T}}}\widehat{\phi}_{inc}^{\pm\vec{K}}(cell_0)$ are $2N_s(l_{max}+1)\times 1$ column vectors given by:

$$\widehat{\widehat{\mathbf{T}}}\widehat{\psi}_{\pm\vec{K}}(cell_0) = \begin{pmatrix} \widehat{\widehat{\mathbf{T}}}\widehat{\psi}_{\pm\vec{K}}(\vec{r}_{1,0}) \\ \widehat{\widehat{\mathbf{T}}}\widehat{\psi}_{\pm\vec{K}}(\vec{r}_{2,0}) \\ \widehat{\widehat{\mathbf{T}}}\widehat{\psi}_{\pm\vec{K}}(\vec{r}_{3,0}) \\ \vdots \\ \widehat{\widehat{\mathbf{T}}}\widehat{\psi}_{\pm\vec{K}}(\vec{r}_{N_s,0}) \end{pmatrix}_{\pm\vec{K}}, \widehat{\widehat{\mathbf{T}}}\widehat{\phi}_{inc}^{\pm\vec{K}}(cell_0) = \begin{pmatrix} \widehat{\widehat{\mathbf{T}}}\widehat{\phi}_{inc}^{\pm\vec{K}}(\vec{r}_{1,0}) \\ \widehat{\widehat{\mathbf{T}}}\widehat{\phi}_{inc}^{\pm\vec{K}}(\vec{r}_{2,0}) \\ \widehat{\widehat{\mathbf{T}}}\widehat{\phi}_{inc}^{\pm\vec{K}}(\vec{r}_{3,0}) \\ \vdots \\ \widehat{\widehat{\mathbf{T}}}\widehat{\phi}_{inc}^{\pm\vec{K}}(\vec{r}_{N_s,0}) \end{pmatrix}_{\pm\vec{K}} \tag{8}$$

where for $m = \{1,\ldots,N_s\}$, $\widehat{\widehat{\mathbf{T}}}\widehat{\psi}_{\pm\vec{K}}(\vec{r}_{m,0})$ and $\widehat{\widehat{\mathbf{T}}}\widehat{\phi}_{inc}^{\pm\vec{K}}(\vec{r}_{m,0})$ are $2(l_{max}+1)\times 1$ column vectors given by:

$$\widehat{\widehat{\mathbf{T}}}\widehat{\psi}_{\pm\vec{K}}(\vec{r}_{m,0}) = \begin{pmatrix} \psi_{\pm\vec{K}}(\vec{r}_{m,0}) \\ \widehat{T}_{1,\pm\vec{K}}\psi_{\pm\vec{K}}(\vec{r}_{m,0}) \\ \widehat{T}_{2,\pm\vec{K}}\psi_{\pm\vec{K}}(\vec{r}_{m,0}) \\ \vdots \\ \widehat{T}_{l_{max},\pm\vec{K}}\psi_{\pm\vec{K}}(\vec{r}_{m,0}) \end{pmatrix}_{\pm\vec{K}}, \widehat{\widehat{\mathbf{T}}}\widehat{\phi}_{inc}^{\pm\vec{K}}(\vec{r}_{m,0}) = \sqrt{\frac{k_1}{2\nu_F k_{X1}}} e^{i\vec{k}_1\cdot\vec{r}_{m,0}} \begin{pmatrix} 1 \\ \pm e^{i\theta_{\vec{k}_1}} \\ e^{-i\theta_k} \\ \pm e^{2i\theta_{\vec{k}_1}} \\ e^{-2i\theta_{\vec{k}_1}} \\ \pm e^{3i\theta_{\vec{k}_1}} \\ \vdots \\ e^{-l_{max}i\theta_{\vec{k}_1}} \\ \pm e^{(l_{max}+1)i\theta_{\vec{k}_1}} \end{pmatrix}_{\pm\vec{K}} \tag{9}$$

and $\widehat{\widehat{\mathbf{TG}}}_{\pm\vec{K}}(k_1 d, k_{Y1} d)$ is a $2N_s(l_{max}+1)\times 2N_s(l_{max}+1)$ matrix given by:

$$\widehat{\widehat{\mathbf{TG}}}_{\pm\vec{K}}(k_1 d, k_{Y1} d) = \begin{pmatrix} \widehat{\widehat{\mathbf{TD}}}^{\pm\vec{K}}_{(1,1)}(k_1 d, k_{Y1} d) & \widehat{\widehat{\mathbf{TG}}}^{\pm\vec{K}}_{(1,2)}(k_1 d, k_{Y1} d) & \ldots & \widehat{\widehat{\mathbf{TG}}}^{\pm\vec{K}}_{(1,N_s)}(k_1 d, k_{Y1} d) \\ \widehat{\widehat{\mathbf{TG}}}^{\pm\vec{K}}_{(2,1)}(k_1 d, k_{Y1} d) & \widehat{\widehat{\mathbf{TD}}}^{\pm\vec{K}}_{(2,2)}(k_1 d, k_{Y1} d) & \ldots & \widehat{\widehat{\mathbf{TG}}}^{\pm\vec{K}}_{(2,N_s)}(k_1 d, k_{Y1} d) \\ \vdots & \vdots & \ddots & \vdots \\ \widehat{\widehat{\mathbf{TG}}}^{\pm\vec{K}}_{(N_s,1)}(k_1 d, k_{Y1} d) & \widehat{\widehat{\mathbf{TG}}}^{\pm\vec{K}}_{(N_s,2)}(k_1 d, k_{Y1} d) & \ldots & \widehat{\widehat{\mathbf{TD}}}^{\pm\vec{K}}_{(N_s,N_s)}(k_1 d, k_{Y1} d) \end{pmatrix}_{\pm\vec{K}} \tag{10}$$

where for $m \neq n$:

$$\widehat{\widehat{\mathbf{TG}}}^{\pm\vec{K}}_{(m,n)}(k_1 d, k_{Y1} d) = \begin{pmatrix} s_{n,0}\tilde{G}^{(m,n),\pm\vec{K}}_{0,0}(k_1 d, k_{Y1} d) & s_{n,1}\tilde{G}^{(m,n),\pm\vec{K}}_{0,1}(k_1 d, k_{Y1} d) & \ldots & s_{n,l_{max}}\tilde{G}^{(m,n),\pm\vec{K}}_{0,l_{max}}(k_1 d, k_{Y1} d) \\ s_{n,0}\tilde{G}^{(m,n),\pm\vec{K}}_{1,0}(k_1 d, k_{Y1} d) & s_{n,1}\tilde{G}^{(m,n),\pm\vec{K}}_{1,1}(k_1 d, k_{Y1} d) & \ldots & s_{n,l_{max}}\tilde{G}^{(m,n),\pm\vec{K}}_{1,l_{max}}(k_1 d, k_{Y1} d) \\ \vdots & \vdots & \ddots & \vdots \\ s_{n,0}\tilde{G}^{(m,n),\pm\vec{K}}_{l_{max},0}(k_1 d, k_{Y1} d) & s_{n,1}\tilde{G}^{(m,n),\pm\vec{K}}_{l_{max},1}(k_1 d, k_{Y1} d) & \ldots & s_{n,l_{max}}\tilde{G}^{(m,n),\pm\vec{K}}_{l_{max},l_{max}}(k_1 d, k_{Y1} d) \end{pmatrix}_{\pm\vec{K}} \tag{11}$$

with

$$\begin{aligned} \tilde{G}^{(m,p),\pm\vec{K}}_{l',l}(k_1 d, k_{Y1} d) &= i^{l+l'} \sum_{n=-\infty}^{\infty} e^{ik_{Y1}nd} \begin{pmatrix} (-1)^{l'} H^{(1)}_{l-l'}(k_1 r_n^{(m,p)}) e^{i(l-l')\theta_{\vec{r}_n^{(m,p)}}} & \pm i H^{(1)}_{l+l'+1}(k_1 r_n^{(m,p)}) e^{-i(l+l'+1)\theta_{\vec{r}_n^{(m,p)}}} \\ \pm i H^{(1)}_{l+l'+1}(k_1 r_n^{(m,p)}) e^{i(l+l'+1)\theta_{\vec{r}_n^{(m,p)}}} & (-1)^{l} H^{(1)}_{l'-l}(k_1 r_n^{(m,p)}) e^{i(l'-l)\theta_{\vec{r}_n^{(m,p)}}} \end{pmatrix}_{\pm\vec{K}} \\ &= \sum_{n=-\infty}^{\infty} e^{ik_{Y1}nd} \begin{pmatrix} \widehat{L}_{+}^{l-l'}\left[H_0^{(1)}(k_1 r_n^{(m,p)})\right] & \pm\widehat{L}_{-}^{l+l'+1}\left[H_0^{(1)}(k_1 r_n^{(m,p)})\right] \\ \pm\widehat{L}_{+}^{l+l'+1}\left[H_0^{(1)}(k_1 r_n^{(m,p)})\right] & \widehat{L}_{-}^{l-l'}\left[H_0^{(1)}(k_1 r_n^{(m,p)})\right] \end{pmatrix}_{\pm\vec{K}} \\ &= \begin{pmatrix} SS_{l-l'}(k_1, k_{Y1}, d, \vec{\Delta}r_{m,p}) & \pm SS_{-(l+l'+1)}(k_1, k_{Y1}, d, \vec{\Delta}r_{m,p}) \\ \pm SS_{l+l'+1}(k_1, k_{Y1}, d, \vec{\Delta}r_{m,p}) & SS_{-(l-l')}(k_1, k_{Y1}, d, \vec{\Delta}r_{m,p}) \end{pmatrix}_{\pm\vec{K}} \end{aligned} \tag{12}$$

where $r_n^{(m,p)} = \left|\vec{\Delta}r_{m,p} - nd\widehat{y}\right|$, and $e^{\pm i\theta_{\vec{r}_n^{(m,p)}}} = (\vec{\Delta}r_{m,p} - nd\widehat{y}) \cdot \frac{\widehat{x}\pm i\widehat{y}}{r_n^{(m,p)}}$.

The diagonal elements of $\widehat{\widehat{\mathbf{TG}}}$ are given by:

$$\widehat{\widehat{\mathbf{TD}}}^{\pm\vec{K}}_{(m,m)}(k_1 d, k_{Y1} d) = \begin{pmatrix} s_{m,0}\tilde{D}^{\pm\vec{K}}_{0,0}(k_1 d, k_{Y1} d) & s_{m,1}\tilde{D}^{\pm\vec{K}}_{0,1}(k_1 d, k_{Y1} d) & \dots & s_{m,l_{max}}\tilde{D}^{\pm\vec{K}}_{0,l_{max}}(k_1 d, k_{Y1} d) \\ s_{m,0}\tilde{D}^{\pm\vec{K}}_{1,0}(k_1 d, k_{Y1} d) & s_{m,1}\tilde{D}^{\pm\vec{K}}_{1,1}(k_1 d, k_{Y1} d) & \dots & s_{m,l_{max}}\tilde{D}^{\pm\vec{K}}_{1,l_{max}}(k_1 d, k_{Y1} d) \\ \vdots & \vdots & \ddots & \vdots \\ s_{m,0}\tilde{D}^{\pm\vec{K}}_{l_{max},0}(k_1 d, k_{Y1} d) & s_{m,1}\tilde{D}^{\pm\vec{K}}_{l_{max},1}(k_1 d, k_{Y1} d) & \dots & s_{m,l_{max}}\tilde{D}^{\pm\vec{K}}_{l_{max},l_{max}}(k_1 d, k_{Y1} d) \end{pmatrix}_{\pm\vec{K}} \quad (13)$$

with

$$\begin{aligned}\tilde{D}^{\pm\vec{K}}_{l',l}(k_1 d, k_{Y1} d) &= i^{l+l'} \sum_{n\neq 0} e^{ik_{Y1}nd} \begin{pmatrix} (-1)^{l'} H^{(1)}_{l-l'}(k_1|n|d) e^{i(l-l')\theta_{0n}} & \pm i H^{(1)}_{l+l'+1}(k_1|n|d) e^{-i(l+l'+1)\theta_{0n}} \\ \pm i H^{(1)}_{l+l'+1}(k_1|n|d) e^{i(l+l'+1)\theta_{0n}} & (-1)^{l} H^{(1)}_{l'-l}(k_1|n|d) e^{i(l'-l)\theta_{0n}} \end{pmatrix}_{\pm\vec{K}} \\ &= \begin{pmatrix} S_{l-l'}(k_1 d, k_{Y1} d) & \pm(-1)^{l+l'+1} S_{l+l'+1}(k_1 d, k_{Y1} d) \\ \pm S_{l+l'+1}(k_1 d, k_{Y1} d) & (-1)^{l-l'} S_{l-l'}(k_1 d, k_{Y1} d) \end{pmatrix}_{\pm\vec{K}}\end{aligned} \quad (14)$$

where $e^{\pm i\theta_{0n}} = \mp i\frac{n}{|n|}$ and

$$S_l(k_1 d, k_{Y1} d) = \sum_{n=1}^{\infty} H^{(1)}_l(k_1 n d)\left(e^{ik_{Y1}nd} + (-1)^l e^{-ik_{Y1}nd}\right) \quad (15)$$

The individual matrix elements in Eq. (12) can be computed one of two ways, depending upon $\vec{\Delta}r_{m,j} = r_m - \vec{r}_j$. When $\left|\widehat{x}\cdot\vec{\Delta}r_{m,j}\right| \geq \frac{d}{2}$, $SS_l(k_1, k_{Y1}, d, \vec{\Delta}r_{m,p})$ in Eq. (12) can be efficiently calculated using a plane wave expansion[4]:

$$\begin{aligned} SS_l(k_1, k_{Y1}, d, \vec{\Delta}r_{m,p}) &= \sum_{n=-\infty}^{\infty} \widehat{L}^{|l|}_{\mathrm{sign}(l)}\left[H^{(1)}_0\left(k_1 r_n^{(m,p)}\right)\right] e^{ik_{Y1}nd} \\ &= \frac{2}{d}\sum_{n=-\infty}^{\infty} \frac{e^{i\left(k^{(n)}_{Y1}\widehat{y}\cdot\vec{\Delta}r_{m,j} + k^{(n)}_{X1}\left|\widehat{x}\cdot\vec{\Delta}r_{m,j}\right|\right)}}{k^{(n)}_{X1}} \left(\mathrm{sign}\left(\widehat{x}\cdot\vec{\Delta}r_{m,j}\right) e^{i\,\mathrm{sign}(l)\,\mathrm{sign}\left(\widehat{x}\cdot\vec{\Delta}r_{m,j}\right)\theta_{\vec{k}_1^{(n)}}}\right)^{|l|} \end{aligned} \quad (16)$$

where either $k^{(n)}_{Y1} = k_{Y1} + \frac{2\pi n}{d}$, and $k^{(n)}_{X1} = \sqrt{k_1^2 - \left(k^{(n)}_{Y1}\right)^2}$ and $e^{\pm i\theta_{\vec{k}_1^{(n)}}} = \frac{k^{(n)}_{X1} \pm i k^{(n)}_{Y1}}{k_1}$ for $k_1 \geq k^{(n)}_{Y1}$ or $k^{(n)}_{X1} = i\sqrt{\left(k^{(n)}_{Y1}\right)^2 - k_1^2}$ and $e^{\pm i\theta_{\vec{k}_1^{(n)}}} = i\frac{k^{(n)}_{X1} \pm k^{(n)}_{Y1}}{k_1}$ for $k_1 \leq k^{(n)}_{Y1}$. Note that $k^{(n)}_{X1}$ will be real for $n \in \mathcal{N} = \{\mathcal{N}_{\min}, \cdots, \mathcal{N}_{\max}\}$ where $\mathcal{N}_{\min} = \left\{\frac{-(k_1+k_{Y1})d}{2\pi}\right\}_+$ and $\mathcal{N}_{\max} = \left\{\frac{(k_1-k_{Y1})d}{2\pi}\right\}_-$, where $\{z\}_+$ corresponds to the smallest integer greater than z, and $\{z\}_-$ corresponds to the largest integer less than z. The requirement that $\left|\widehat{x}\cdot\vec{\Delta}r_{m,n}\right| \geq \frac{d}{2}$ insures that the summation in Eq. (16) can be safely approximated using only a finite number of evanescent waves.

For $|\vec{\Delta r}_{m,p}| \leq \frac{d}{2}$, the convergence of Eq. (16) is slow due to the necessity of including a large number of evanescent waves in the calculation, particularly when $|\vec{\Delta r}_{m,p}| \approx 0$. In this case, the individual matrix elements in Eq. (12) can be calculated using Graf's theorem[4, 5]:

$$SS_l(k_1, k_{Y1}, d, \vec{\Delta r}_{m,p}) = i^l H_l^{(1)}\left(k_1 \left|\vec{\Delta r}_{m,p}\right|\right) e^{il\theta_{\vec{\Delta r}_{m,p}}} + \sum_{n=-\infty}^{\infty} S_{|n|}(k_1 d, k_{Y1} d) i^{l-|n|} J_{n+l}\left(k_1 \left|\vec{\Delta r}_{m,p}\right|\right) e^{i(n+l)\theta_{\vec{\Delta r}_{m,p}}} \tag{17}$$

In both Eq. (17) and for the matrix elements in Eq. (14), the lattice sum, $S_l(k_1 d, k_{Y1} d)$ in Eq. (15), can be efficiently calculated using [2, 5]:

$$S_l(k_1 d, k_{Y1} d) = \frac{\sqrt{2}}{\pi} e^{-i\left(\frac{\pi}{4} - k_{Y1} d\right)} \int_0^a \mathrm{d}t \frac{\left[\left(t(1-i) + i\sqrt{1+2it^2}\right)^l + \left(t(i-1) + i\sqrt{1+2it^2}\right)^l\right] e^{ik_1 d\sqrt{1+2it^2}}}{\sqrt{1+2it^2}\left(1 - e^{i\left(k_1 d\sqrt{1+2it^2} + k_{Y1} d\right)}\right)} + (-1)^l \frac{\sqrt{2}}{\pi} e^{-i\left(\frac{\pi}{4} + k_{Y1} d\right)} \int_0^a \mathrm{d}t \frac{\left[\left(t(1-i) + i\sqrt{1+2it^2}\right)^l + \left(t(i-1) + i\sqrt{1+2it^2}\right)^l\right] e^{ik_1 d\sqrt{1+2it^2}}}{\sqrt{1+2it^2}\left(1 - e^{i\left(k_1 d\sqrt{1+2it^2} - k_{Y1} d\right)}\right)} \tag{18}$$

In this work, the expression for $S_l(k_1 d, k_{Y1} d)$ in Eq. (18) was numerically integrated using MATLAB[6], where the upper limit of the integrals in Eq. (18) was chosen to be $a = 4000$.

In this case, $\psi_{\pm\vec{K}}(\vec{r})$ in Eq. (2) can be written as a sum over plane and evanescent waves as:

$$\psi_{\pm\vec{K}}(\vec{r}) = \phi_{inc}^{\pm\vec{K}}(\vec{r}) + \sum_{n=-\infty}^{\infty} \sum_{m=1}^{N_s} \sum_{l=0}^{l_{max}} \frac{2s_{m,l}}{d} \frac{e^{i(k_{Y1}^{(n)}(y - \hat{y}\cdot\vec{r}_m) + k_{X1}^{(n)}|x - \hat{x}\cdot\vec{r}_m|)}}{k_{X1}^{(n)}} \left(\mathrm{sign}\left(x - \hat{x}\cdot\vec{r}_m\right)\right)^l \times \begin{pmatrix} e^{i\mathrm{sign}(x-\hat{x}\cdot\vec{r}_m) l\theta_{\vec{k}_1^{(n)}}} & \pm\mathrm{sign}\left(x - \hat{x}\cdot\vec{r}_m\right) e^{-i\mathrm{sign}(x-\hat{x}\cdot\vec{r}_m)(l+1)\theta_{\vec{k}_1^{(n)}}} \\ \pm\mathrm{sign}\left(x - \hat{x}\cdot\vec{r}_m\right) e^{i\mathrm{sign}(x-\hat{x}\cdot\vec{r}_m)(l+1)\theta_{\vec{k}_1^{(n)}}} & e^{-i\mathrm{sign}(x-\hat{x}\cdot\vec{r}_m) l\theta_{\vec{k}_1^{(n)}}} \end{pmatrix}_{\pm\vec{K}} \widehat{T}_{l,\pm\vec{K}} \psi_{\pm\vec{K}}(\vec{r}_m) \tag{19}$$

In Eq. (19), $\psi_{\pm\vec{K}}(\vec{r})$ consists of a series of plane waves for $n \in \mathcal{N}$ that are either transmitted $[x > 0]$ or reflected $[x < 0]$ from the scattering array along with evanescent waves along the $\hat{x}$-direction that are freely propagating along the $\hat{y}$-direction for $n \notin \mathcal{N}$.

For $x \gg d$, the transmitted wave function, $\psi_{\pm\vec{K},T}(\vec{r})$, can be written as:

$$\psi_{\pm\vec{K},T}(\vec{r}) = \sum_{n\in\mathcal{N}} T_n e^{i\vec{k}_1^{(n)}\cdot\vec{r}} \sqrt{\frac{k_1}{2v_F k_{X1}^{(n)}}} \begin{pmatrix} 1 \\ \pm e^{i\theta_{\vec{k}_1^{(n)}}} \end{pmatrix}_{\pm\vec{K}} \tag{20}$$

where the sum in Eq. (20) is over all open channels, $n \in \mathcal{N}$, with the transmission coefficient for the nth channel given by:

$$
\begin{aligned}
T_n &= \delta_{n0} + \sum_{m=1}^{N_s}\sum_{l=0}^{l_{max}} \frac{s_{m,l} e^{-i\vec{k}_1^{(n)}\cdot\vec{r}_{m,0}}}{d}\sqrt{\frac{2v_F}{k_1 k_{X1}^{(n)}}}\left[\begin{pmatrix} 1 \\ \pm e^{i\theta_{\vec{k}_1^{(n)}}}\end{pmatrix}_{\pm\vec{K}}\right]^{\dagger}\begin{pmatrix} e^{il\theta_{\vec{k}_1^{(n)}}} & \pm e^{-i(l+1)\theta_{\vec{k}_1^{(n)}}} \\ \pm e^{i(l+1)\theta_{\vec{k}_1^{(n)}}} & e^{-il\theta_{\vec{k}_1^{(n)}}}\end{pmatrix}_{\pm\vec{K}} \widehat{T}_{l,\pm\vec{K}}\psi_{\pm\vec{K}}(\vec{r}_{m,0}) \\
&= \delta_{n0} + e^{-\frac{i}{2}\theta_{\vec{k}_1^{(n)}}}\sum_{m=1}^{N_s}\sum_{l=0}^{l_{max}} \frac{2s_{m,l} e^{-i\vec{k}_1^{(n)}\cdot\vec{r}_{m,0}}}{d}\sqrt{\frac{2v_F}{k_1 k_{X1}^{(n)}}}\left[\begin{pmatrix} e^{i\left(l+\frac{1}{2}\right)\theta_{\vec{k}_1^{(n)}}} \\ \pm e^{-i\left(l+\frac{1}{2}\right)\theta_{\vec{k}_1^{(n)}}}\end{pmatrix}_{\pm\vec{K}}\right]^{\mathrm{T}} \widehat{T}_{l,\pm\vec{K}}\psi_{\pm\vec{K}}(\vec{r}_{m,0})
\end{aligned} \tag{21}
$$

where δ_{ij} is the Kronecker delta ($\delta_{ij} = 0$ for $i \neq j$ and $\delta_{ij} = 1$ for $i = j$). From Eq. (21), the transmission coefficients can be written as $T_n = e^{-\frac{i}{2}\theta_{\vec{k}^{(n)}1}}|T_n|$ for $n \neq 0$ and $T_0 = 1 + e^{-\frac{i}{2}\theta_{\vec{k}_1^{(0)}}} t_0$.

Likewise, the reflected wave function ($x \ll -d$), $\psi_{\pm\vec{K},R}(\vec{r})$, is given by:

$$
\psi_{\pm\vec{K},R}(\vec{r}) = \sum_{n\in\mathcal{N}} R_n e^{i\overline{\vec{k}_1^{(n)}}\cdot\vec{r}}\sqrt{\frac{k_1}{2v_F k_{X1}^{(n)}}}\begin{pmatrix} 1 \\ \mp e^{-i\theta_{\vec{k}_1^{(n)}}}\end{pmatrix}_{\pm\vec{K}} \tag{22}
$$

where $\overline{\vec{k}_1^{(n)}} = k_{Y1}^{(n)}\widehat{y} - k_{X1}^{(n)}\widehat{x}$, and

$$
\begin{aligned}
R_n &= \sum_{m=1}^{N_s}\sum_{l=0}^{l_{max}} \frac{(-1)^l s_{m,l} e^{-i\overline{\vec{k}_1^{(n)}}\cdot\vec{r}_{m,0}}}{d}\sqrt{\frac{2v_F}{k_1 k_{X1}^{(n)}}}\left[\begin{pmatrix} 1 \\ \mp e^{i\theta_{\vec{k}_1^{(n)}}}\end{pmatrix}_{\pm\vec{K}}\right]^{\dagger}\begin{pmatrix} e^{-il\theta_{\vec{k}_1^{(n)}}} & \mp e^{i(l+1)\theta_{\vec{k}_1^{(n)}}} \\ \mp e^{-i(l+1)\theta_{\vec{k}_1^{(n)}}} & e^{il\theta_{\vec{k}_1^{(n)}}}\end{pmatrix}_{\pm\vec{K}} \widehat{T}_{l,\pm\vec{K}}\psi_{\pm\vec{K}}(\vec{r}_{m,0}) \\
&= e^{\frac{i}{2}\theta_{\vec{k}_1^{(n)}}}\sum_{m=1}^{N_s}\sum_{l=0}^{l_{max}} \frac{(-1)^l 2s_{m,l} e^{-i\overline{\vec{k}_1^{(n)}}\cdot\vec{r}_{m,0}}}{d}\sqrt{\frac{2v_F}{k_1 k_{X1}^{(n)}}}\left[\begin{pmatrix} e^{-i\left(l+\frac{1}{2}\right)\theta_{\vec{k}_1^{(n)}}} \\ \mp e^{i\left(l+\frac{1}{2}\right)\theta_{\vec{k}_1^{(n)}}}\end{pmatrix}_{\pm\vec{K}}\right]^{\mathrm{T}} \widehat{T}_{l,\pm\vec{K}}\psi_{\pm\vec{K}}(\vec{r}_{m,0}) \\
&= e^{\frac{i}{2}\theta_{\vec{k}_1^{(n)}}}|R_n|
\end{aligned} \tag{23}
$$

The transmission and reflection coefficients satisfy the unitarity condition, $\sum_{n\in\mathcal{N}} |R_n|^2 + |T_n|^2 = 1$.

II. CALCULATIONS OF OF THE SCATTERING STATES FROM AN INFINITE ONE-DIMENSIONAL ARRAY OF LOCALIZED SCATTERERS IN A TWO-DIMENSIONAL ELECTRON GAS (2DEG), $\psi^{\mathrm{ac}}(\vec{r})$

In a 2DEG, consider an electron wave with effective mass m^*, energy $E = \sqrt{\frac{\hbar^2 k_1^2}{2m^*}}$, wave vector $\vec{k}_1 = k_{X1}\widehat{x} + k_{Y1}\widehat{y} = k_1\cos(\theta_{\vec{k}_1})\widehat{x} + k_1\sin(\theta_{\vec{k}_1})\widehat{y}$ and normalized to unit flux along the $\widehat{x}-$direction, $\phi_{inc}^{\mathrm{ac}}(\vec{r}) = \sqrt{\frac{m^*}{\hbar k_{X1}}}e^{-i\vec{k}_1\cdot\vec{r}}$, that is incident to a periodic scattering potential $\widehat{V}(\vec{r}) = \sum_{n=-\infty}^{\infty}\sum_{m=1}^{N_s} V_m \Theta_{r_{sm}}(\vec{r} - \vec{r}_{m,n})$ as depicted in Figure 1.

In this case, the total wave function, $\psi^{\rm ac}(\vec{r})$, can be written as [for $x \neq 0$]:

$$\begin{aligned}\psi^{\rm ac}(\vec{r}) &= \phi^{\rm ac}_{inc}(\vec{r}) + \sum_{m=1}^{N_s}\sum_{n=-\infty}^{\infty} e^{ik_{Y1}nd} \sum_{l=-l_{max}}^{l_{max}} s^{ac}_{m,l}\widehat{L}^{|l|}_{\mathrm{sign}(l)}\left[H_0^{(1)}(k_1|\vec{r}-\vec{r}_{m,n}|)\right]\widehat{L}^{|l|}_{-\mathrm{sign}(l)}\left[\psi^{\rm ac}(\vec{r}_{m,0})\right] \\ &= \phi^{\rm ac}_{inc}(\vec{r}) + \sum_{m=1}^{N_s}\sum_{l=-l_{max}}^{l_{max}}\sum_{n=-\infty}^{\infty}\frac{2s^{ac}_l}{d}\frac{e^{i(k^{(n)}_{Y1}(y-\widehat{y}\cdot\vec{r}_{m,0})+k^{(n)}_{X1}|x-\widehat{x}\cdot\vec{r}_{m,0}|)}}{k^{(n)}_{X1}}(\mathrm{sign}(x-\widehat{x}\cdot\vec{r}_{m,0}))^l e^{i\mathrm{sign}(x-\widehat{x}\cdot\vec{r}_{m,0})l\theta_{\vec{k}^{(n)}_1}}\widehat{L}^{|l|}_{-\mathrm{sign}(l)}\left[\psi^{\rm ac}(\vec{r}_{m,0})\right]\end{aligned} \tag{24}$$

where $2l_{max}+1$ partial waves are included in the calculation of $\psi^{\rm ac}(\vec{r})$ in Eq. (24), and $s^{\rm ac}_{m,l}$ is the scattering amplitude of the lth partial wave from m^{th} scatterer, which is given for cylindrically symmetric scatterers as:

$$s^{ac}_{m,l} = \frac{J_l(k_1 r_{sm})J_{l+1}(k_{2,m}r_{sm}) - J_l(k_{2,m}r_{sm})J_{l+1}(k_1 r_{sm})}{k_1 H^{(1)}_{l+1}(k_1 r_{sm})J_l(k_{2,m}r_{sm}) - k_{2,m}J_{l+1}(k_{2,m}r_{sm})H^{(1)}_l(k_1 r_{sm})} \tag{25}$$

where $k_{2,m} = \sqrt{\frac{2m^*(E-V_m)}{\hbar^2}}$ for $E \geq V_m$ or $k_{2,m} = i\sqrt{\frac{2m^*|E-V_m|}{\hbar^2}}$ for $E < V_m$. Note that $s^{ac}_{m,l} = s^{ac}_{m,-l}$ in Eq. (25).

Knowledge of $\widehat{L}^{|l|}_{\pm}[\psi^{\rm ac}(\vec{r}_{m,0})]$ for $m \in [1, ..., N_s]$ and $l \in [0, ..., l_{max}]$ completely determines $\psi^{\rm ac}(\vec{r})$ in Eq. (24); these can be determined self-consistently from the following:

$$\begin{aligned}\widehat{L}^{|l'|}_{\pm}[\psi^{\rm ac}(\vec{r}_{m,0})] &= \widehat{L}^{|l'|}_{\pm}[\phi^{\rm ac}(\vec{r}_{m,0})] + \sum_{l=-l_{max}}^{l_{max}}\sum_{n\neq 0} e^{ik_{Y1}nd}s^{ac}_{m,l}\widehat{L}^{|l\pm l'|}_{\mathrm{sign}(l\pm l')}\left[H^{(1)}_0(k_1|n|d)\right]\widehat{L}^{|l|}_{-\mathrm{sign}(l)}\left[\psi^{\rm ac}(\vec{r}_{m,0})\right] \\ &+ \sum_{p\neq m}\sum_{l=-l_{max}}^{l_{max}}\sum_{n=-\infty}^{\infty} e^{ik_{Y1}nd}s^{ac}_{m,l}\widehat{L}^{|l\pm l'|}_{\mathrm{sign}(l\pm l')}\left[H^{(1)}_0(k_1|\vec{\Delta r}_{m,p}-nd\widehat{y}|)\right]\widehat{L}^{|l|}_{-\mathrm{sign}(l)}\left[\psi^{\rm ac}(\vec{r}_{p,0})\right]\end{aligned} \tag{26}$$

Eq. (26) gives a total of $N_s(2l_{max}+1)$ sets of equations, which can be written compactly as:

$$\left(\widehat{\widehat{\mathbf{1}}} - \widehat{\widehat{\mathbf{TG}}}^{\rm ac}(k_1 d, k_{Y1}d)\right)\widehat{\widehat{\mathbf{T}}}\widehat{\psi}^{\rm ac}(cell_0) = \widehat{\widehat{\mathbf{T}}}\widehat{\phi}^{\rm ac}_{inc}(cell_0) \tag{27}$$

where $\widehat{\widehat{\mathbf{1}}}$ is a $N_s(2l_{max}+1)\times N_s(2l_{max}+1)$ identity matrix, $\widehat{\widehat{\mathbf{T}}}\widehat{\psi}^{\rm ac}(cell_0)$ and $\widehat{\widehat{\mathbf{T}}}\widehat{\phi}^{\rm ac}(cell_0)$ are $N_s(2l_{max}+1)\times 1$ column vectors given by:

$$\widehat{\widehat{\mathbf{T}}}\widehat{\psi}^{\rm ac}(cell_0) = \begin{pmatrix}\widehat{\widehat{\mathbf{T}}}\widehat{\psi}^{\rm ac}(\vec{r}_{1,0}) \\ \widehat{\widehat{\mathbf{T}}}\widehat{\psi}^{\rm ac}(\vec{r}_{2,0}) \\ \widehat{\widehat{\mathbf{T}}}\widehat{\psi}^{\rm ac}(\vec{r}_{3,0}) \\ \vdots \\ \widehat{\widehat{\mathbf{T}}}\widehat{\psi}^{\rm ac}(\vec{r}_{N_s,0})\end{pmatrix}, \widehat{\widehat{\mathbf{T}}}\widehat{\phi}^{\rm ac}_{inc}(cell_0) = \begin{pmatrix}\widehat{\widehat{\mathbf{T}}}\widehat{\phi}^{\rm ac}_{inc}(\vec{r}_{1,0}) \\ \widehat{\widehat{\mathbf{T}}}\widehat{\phi}^{\rm ac}_{inc}(\vec{r}_{2,0}) \\ \widehat{\widehat{\mathbf{T}}}\widehat{\phi}^{\rm ac}_{inc}(\vec{r}_{3,0}) \\ \vdots \\ \widehat{\widehat{\mathbf{T}}}\widehat{\phi}^{\rm ac}_{inc}(\vec{r}_{N_s,0})\end{pmatrix}_{\pm\vec{K}} \tag{28}$$

where for $m=\{1,\ldots,N_s\}$, $\widehat{\widehat{\mathbf{T}}}\widehat{\psi}^{\mathrm{ac}}(\vec{r}_{m,0})$ and $\widehat{\widehat{\mathbf{T}}}\widehat{\phi}^{\mathrm{ac}}_{inc}(\vec{r}_{m,0})$ are $(2l_{max}+1)\times 1$ column vectors given by:

$$\widehat{\widehat{\mathbf{T}}}\widehat{\psi}^{\mathrm{ac}}(\vec{r}_{m,0})=\begin{pmatrix}\widehat{L}_{-}^{l_{max}}\left[\psi^{\mathrm{ac}}(\vec{r}_{m,0})\right]\\ \widehat{L}_{-}^{l_{max}-1}\left[\psi^{\mathrm{ac}}(\vec{r}_{m,0})\right]\\ \vdots\\ \psi^{\mathrm{ac}}(\vec{r}_{m,0})\\ \vdots\\ \widehat{L}_{+}^{l_{max}-1}\left[\psi^{\mathrm{ac}}(\vec{r}_{m,0})\right]\\ \widehat{L}_{+}^{l_{max}}\left[\psi^{\mathrm{ac}}(\vec{r}_{m,0})\right]\end{pmatrix},\widehat{\widehat{\mathbf{T}}}\widehat{\phi}^{\mathrm{ac}}_{inc}(\vec{r}_{m,0})=e^{i\vec{k}_1\cdot\vec{r}_{m,0}}\sqrt{\frac{m}{\hbar k_{X1}}}\begin{pmatrix}e^{-il_{max}\theta_{\vec{k}_1}}\\ e^{-i(l_{max}-1)\theta_{\vec{k}_1}}\\ \vdots\\ 1\\ \vdots\\ e^{i(l_{max}-1)\theta_{\vec{k}_1}}\\ e^{il_{max}\theta_{\vec{k}_1}}\end{pmatrix} \tag{29}$$

In Eq. (27), $\widehat{\widehat{\mathbf{TG}}}^{\mathrm{ac}}(k_1d,k_{Y1}d)$ is a $N_s(2l_{max}+1)\times N_s(2l_{max}+1)$ matrix given by:

$$\widehat{\widehat{\mathbf{TG}}}^{\mathrm{ac}}(k_1d,k_{Y1}d)=\begin{pmatrix}\widehat{\widehat{\mathbf{TD}}}^{\mathrm{ac}}_{(1,1)}(k_1d,k_{Y1}d) & \widehat{\widehat{\mathbf{TG}}}^{\mathrm{ac}}_{(1,2)}(k_1d,k_{Y1}d) & \ldots & \widehat{\widehat{\mathbf{TG}}}^{\mathrm{ac}}_{(1,N_s)}(k_1d,k_{Y1}d)\\ \widehat{\widehat{\mathbf{TG}}}^{\mathrm{ac}}_{(2,1)}(k_1d,k_{Y1}d) & \widehat{\widehat{\mathbf{TD}}}^{\mathrm{ac}}_{(2,2)}(k_1d,k_{Y1}d) & \ldots & \widehat{\widehat{\mathbf{TG}}}^{\mathrm{ac}}_{(2,N_s)}(k_1d,k_{Y1}d)\\ \vdots & \vdots & \ddots & \vdots\\ \widehat{\widehat{\mathbf{TG}}}^{\mathrm{ac}}_{(N_s,1)}(k_1d,k_{Y1}d) & \widehat{\widehat{\mathbf{TG}}}^{\mathrm{ac}}_{(N_s,2)}(k_1d,k_{Y1}d) & \ldots & \widehat{\widehat{\mathbf{TD}}}^{\mathrm{ac}}_{(N_s,N_s)}(k_1d,k_{Y1}d)\end{pmatrix} \tag{30}$$

where for $m\neq n$

$$\widehat{\widehat{\mathbf{TG}}}^{\mathrm{ac}}_{(m,n)}=\begin{pmatrix}s^{ac}_{n,l_{max}}SS_0(k_1,k_{Y1},d,\vec{\Delta}r_{m,n}) & s^{ac}_{n,l_{max}-1}SS_{-1}(k_1,k_{Y1},d,\vec{\Delta}r_{m,n}) & \ldots & s^{ac}_{-(l_{max}-1)}SS_{-2l_{max}+1}(k_1,k_{Y1},d,\vec{\Delta}r_{m,n}) & s^{ac}_{-l_{max}}SS_{-2l_{max}}(k_1,k_{Y1},d,\vec{\Delta}r_{m,n})\\ s^{ac}_{n,l_{max}}SS_1(k_1,k_{Y1},d,\vec{\Delta}r_{m,n}) & s^{ac}_{n,l_{max}-1}SS_0(k_1,k_{Y1},d,\vec{\Delta}r_{m,n}) & \ldots & s^{ac}_{-(l_{max}-1)}SS_{-2l_{max}+2}(k_1,k_{Y1},d,\vec{\Delta}r_{m,n}) & s^{ac}_{-l_{max}}SS_{-2l_{max}+1}(k_1,k_{Y1},d,\vec{\Delta}r_{m,n})\\ \vdots & \vdots & \ddots & \vdots & \vdots\\ s^{ac}_{n,l_{max}}SS_{2l_{max}-1}(k_1,k_{Y1},d,\vec{\Delta}r_{m,n}) & s^{ac}_{n,l_{max}-1}SS_{2l_{max}-2}(k_1,k_{Y1},d,\vec{\Delta}r_{m,n}) & \ldots & s^{ac}_{-(l_{max}-1)}SS_0(k_1,k_{Y1},d,\vec{\Delta}r_{m,n}) & s^{ac}_{-l_{max}}SS_{-1}(k_1,k_{Y1},d,\vec{\Delta}r_{m,n})\\ s^{ac}_{n,l_{max}}SS_{2l_{max}}(k_1,k_{Y1},d,\vec{\Delta}r_{m,n}) & s^{ac}_{n,l_{max}-1}SS_{2l_{max}-1}(k_1,k_{Y1},d,\vec{\Delta}r_{m,n}) & \ldots & s^{ac}_{-(l_{max}-1)}SS_1(k_1,k_{Y1},d,\vec{\Delta}r_{m,n}) & s^{ac}_{-l_{max}}SS_0(k_1,k_{Y1},d,\vec{\Delta}r_{m,n})\end{pmatrix} \tag{31}$$

The diagonal elements of $\widehat{\widehat{\mathbf{TG}}}^{\mathrm{ac}}$ are given by:

$$\widehat{\widehat{\mathbf{TD}}}^{\mathrm{ac}}_{(m,m)}(k_1d,k_{Y1}d)=\begin{pmatrix}s^{ac}_{m,l_{max}}S_0(k_1d,k_{Y1}d) & s^{ac}_{m,l_{max}-1}S_{-1}(k_1d,k_{Y1}d) & \ldots & s^{ac}_{m,l_{max}-1}S_{-2l_{max}+1}(k_1d,k_{Y1}d) & s^{ac}_{m,l_{max}}S_{-2l_{max}}(k_1d,k_{Y1}d)\\ s^{ac}_{m,l_{max}}S_1(k_1d,k_{Y1}d) & s^{ac}_{m,l_{max}-1}S_0(k_1d,k_{Y1}d) & \ldots & s^{ac}_{m,l_{max}-1}S_{-2l_{max}+2}(k_1d,k_{Y1}d) & s^{ac}_{m,l_{max}}S_{-2l_{max}+1}(k_1d,k_{Y1}d)\\ \vdots & \vdots & \ddots & \vdots & \vdots\\ s^{ac}_{m,l_{max}}S_{2l_{max}-1}(k_1d,k_{Y1}d) & s^{ac}_{m,l_{max}-1}S_{2l_{max}-2}(k_1d,k_{Y1}d) & \ldots & s^{ac}_{m,l_{max}-1}S_0(k_1d,k_{Y1}d) & s^{ac}_{m,l_{max}}S_{-1}(k_1d,k_{Y1}d)\\ s^{ac}_{m,l_{max}}S_{2l_{max}}(k_1d,k_{Y1}d) & s^{ac}_{m,l_{max}-1}S_{2l_{max}-1}(k_1d,k_{Y1}d) & \ldots & s^{ac}_{m,l_{max}-1}S_1(k_1d,k_{Y1}d) & s^{ac}_{m,l_{max}}S_0(k_1d,k_{Y1}d)\end{pmatrix} \tag{32}$$

where $S_l(k_1d,k_{Y1}d)=(-1)^lS_{-l}(k_1d,k_{Y1}d)$ and is given in Eq. (15).

Finally, the transmitted and reflected wave functions, $\psi^{\mathrm{ac}}_T(\vec{r})$ for $x>0$ and $\psi^{\mathrm{ac}}_R(\vec{r})$ for $x<0$, are determined from Eq. (24):

$$\begin{aligned}\psi^{\mathrm{ac}}_T(\vec{r})&=\sum_{n\in\mathcal{N}}T^{\mathrm{ac}}_n e^{i\vec{k}_1^{(n)}\cdot\vec{r}}\sqrt{\frac{m^*}{\hbar k^{(n)}_{X1}}}\\ \psi^{\mathrm{ac}}_R(\vec{r})&=\sum_{n\in\mathcal{N}}R^{\mathrm{ac}}_n e^{i\overline{\vec{k}_1^{(n)}}\cdot\vec{r}}\sqrt{\frac{m^*}{\hbar k^{(n)}_{X1}}}\end{aligned} \tag{33}$$

where the reflection and transmission coefficients satisfy the unitarity condition, $\sum_{n\in\mathcal{N}} |T_n^{\mathrm{ac}}|^2 + |R_n^{\mathrm{ac}}|^2 = 1$, and are given by:

$$T_n^{\mathrm{ac}} = \delta_{n0} + \sum_{m=1}^{N_s} \sum_{l=-l_{max}}^{l_{max}} \frac{2s_{m,l}^{ac} e^{-i\vec{k}_1^{(n)}\cdot\vec{r}_{m,0}}}{d} \sqrt{\frac{m^*}{\hbar k_{X1}^{(n)}}} e^{il\theta_{\vec{k}_1^{(n)}}} \widehat{L}_{-\mathrm{sign}(l)}^{|l|} [\psi^{\mathrm{ac}}(\vec{r}_{m,0})]$$

$$R_n^{\mathrm{ac}} = \sum_{m=1}^{N_s} \sum_{l=-l_{max}}^{l_{max}} \frac{2s_{m,l}^{ac}(-1)^l e^{-i\overline{\vec{k}_1^{(n)}}\cdot\vec{r}_{m,0}}}{d} \sqrt{\frac{m^*}{\hbar k_{X1}^{(n)}}} e^{-il\theta_{\overline{\vec{k}_1^{(n)}}}} \widehat{L}_{-\mathrm{sign}(l)}^{|l|} [\psi^{\mathrm{ac}}(\vec{r}_{m,0})]$$

(34)

[1] J. Y. Vaishnav, J. Q. Anderson, and J. D. Walls, Phys. Rev. B **83**, 165437 (2011).

[2] J. D. Walls and D. Hadad, Sci. Rep. **5**, 8435 (2015).

[3] M. I. Katsnelson and K. S. Novoselov, Sol. State Comm. **143**, 3 (2007).

[4] N. A. Nicorovici, R. C. McPhedran, and R. Petit, Phys. Rev. E **49**, 4563 (1994).

[5] K. Yasumoto and K. Yoshitomi, IEEE Trans. Antennas Propagat. **47**, 1050 (1999).

[6] Mathworks, "Matlab," `http://www.mathworks.com`.
